# Validation of the Persian version of the Positive Mental Health Scale

**DOI:** 10.1186/s12888-021-03487-6

**Published:** 2021-09-27

**Authors:** Azam Naghavi, Tobias Teismann, Zahra Asgari, Razieh Eizadifard, Julia Brailovskaia

**Affiliations:** 1 Department of Counseling, Faculty of Education and Psychology, University of Isfahan, Azadi Sq, Isfahan, 8174673441 Iran; 2 Mental Health Research and Treatment Center, Department of Psychology, Ruhr-Universität Bochum, Massenbergstrasse 9-13, 44787 Bochum, Germany

**Keywords:** Positive mental health, Resilience, Suicide ideation/behavior, Assessment

## Abstract

**Background:**

Positive mental health (PMH) is a factor of far-reaching salutogenetic importance. The present study aimed at validating the Persian version of the Positive Mental Health Scale (PMH-Scale).

**Methods:**

Reliability and validity of the Persian version of the PMH-Scale were established in an Iranian student sample (*N* = 573). Internal consistency, convergent and discriminant validity were investigated, and exploratory factor analysis was conducted. Furthermore, it was assessed how PMH scores moderate the association between depressive symptoms and suicide ideation/behavior.

**Results:**

The Persian version of the PMH-Scale  was shown to have a unidimensional structure with excellent internal consistency, as well as good convergent and divergent validity. PMH differentiated between participants with higher vs. lower suicide risk. Furthermore, PMH proved to moderate the association between depressive symptoms and suicide ideation/behavior.

**Conclusions:**

The results suggest that the PMH-Scale is a brief, reliable, and valid measure of subjective and psychological well-being that can be used in Iranian student samples and research settings.

## Background

Mental health has traditionally been defined as the absence of psychopathology [1]: Individuals were seen as either mentally ill or presumed to be mentally healthy. Meanwhile, it is widely recognized that positive mental health (PMH), i.e., a positive sense of well-being, as well as the capacity to enjoy life and deal with life’s challenges (cf., [1]), and the absence of psychopathology are not the same. It is rather the case that elements of PMH and psychopathology can be present at the same time (“dual factor model of metal health”, e.g., [2]); as such PMH and psychopathology are not opposite ends of a single continuum; rather they represent different but correlated axes [3]. In this view, both PMH and psychopathology are required for complete mental health assessments and should be integrated in research and practice. In fact, various studies point to the fact that PMH is a stronger predictor for the course of mental disorder than markers of psychopathology [4, 5]. Nevertheless, most studies in clinical psychology and psychiatry continue to exclusively focus on negative aspects of mental health. To overcome this deficiency, time-efficient measurement instruments for the assessment of PMH are needed that are equally suitable for use in research and clinical practice.

The Positive Mental Health Scale (PMH-Scale) is such a new, brief, time-efficient and frequently used measure to assess PMH. The scale contains nine items [6] that capture aspects of subjective well-being (“I enjoy my life”), as well as items that capture aspects of psychological well-being, such as environmental mastery (“I manage well to fulfill my needs”) and self-acceptance (“I am in good physical and emotional condition”). The PMH-Scale thus integrates facets from two traditions of well-being research: The hedonic tradition focusing on positive affect and life-satisfaction and the eudemonic tradition focusing on optimal functioning in everyday life [1, 7]. In validation studies of the PMH-Scale a unidimensional structure, good to excellent internal consistency (Cronbach’s alpha = .82–.93), good test-retest reliability (≥ .74) and scalar invariance across samples and over time were demonstrated in research from various countries such as Germany [6], China and Russia [8], Lithuania [9], Pakistan [10], France, Poland, Spain, Sweden, the U.K. and the U.S. [11]. In correlational analyses, the PMH-Scale was negatively associated with self-report measures of depression, anxiety and stress and positively associated with measures of social support, subjective happiness, and life satisfaction [6, 8].

In longitudinal predictor studies, PMH, as assessed with the PMH-Scale [6], was found to be of central importance for the remission of mental disorders in general [4] and anxiety disorders [12–14] as well as suicidal ideation [15] in particular. Furthermore, current level of PMH was shown to be a unique predictor not only of the level of future PMH, but also of the level of future mental health problems [16]. In two recent studies, PMH was identified as a prospective predictor of lower psychological burden experienced by the COVID-19 situation in spring 2020 [17], as well as of greater adherence and acceptance of COVID-19 related restrictions [11]. Finally – and most extensively researched – PMH has been shown to moderate the association between various risk factors (e.g., depression, stress, perceived burdensomeness, entrapment, addictive symptoms) and suicide ideation [18–21]. Furthermore, PMH was found to buffer the association between suicide ideation and suicide attempts [22]. These moderating properties of PMH were shown both in cross-sectional (e.g., [19]) and in longitudinal studies [18, 22–24]. In a first cross-cultural comparison, Siegmann et al. [19] found PMH to buffer the effect of depressive symptoms on suicide ideation in German and Chinese students: Students who reported high levels of PMH showed no increase in suicide ideation even as depression levels increased. Taken together, positive mental health seems to be of extensive salutogenetic importance.

In order to facilitate cross-cultural studies, it is essential to have validated measurement tools in different languages [8–11]. The comparison of Western individualistic cultures with more collectivistic cultures, such as Iran, is of particular interest. With the aim of enabling comparative cross-cultural studies of the conditions and significance of PMH, the present study aimed to examine the factor structure, psychometric properties, and construct validity of the Persian version of the PMH-Scale within an Iranian student sample. Furthermore, it was aimed to investigate, whether PMH, as assessed by the Persian version of the PMH-Scale, buffers the impact of depression on suicide ideation/behavior and as such confers resilience against suicide ideation and behavior in a country with a cultural background different from that in Germany and China [19]. In order to establish comparability with previous studies on the PMH-Scale (e.g., [6, 8–10, 19]) and since suicide ideation/behavior is common in student samples [25], the validation of the Persian PMH-Scale was performed on a student sample.

## Methods

### Participants and procedure

The current study sample comprised 573 participants from Iran (73.1% women; age in years: *M (SD)* = 24.45 (6.65, range: 18–76). All participants were students (63% undergraduates, 37% graduate students) with the following subjects of study: 60.7% liberal arts, 23.6% engineering sciences, 7.2% medical science, 5.8% basic science. Most of the participants were either single (79.2%) or married (19.9%; see [26] for more details).

Data were collected between March 2020 and May 2020 via an online survey. Participants were recruited via participation invitations displayed at social media (i.e., groups on Instagram, WhatsApp, and Telegram). There were no specific requirements for participation that was voluntary and not compensated. The implementation of the present study was approved by the responsible Ethics Committee. All participants were properly instructed and provided their informed consent online. No data sets were excluded. There were no missing data. A priori conducted power analyses (G*Power program, version 3.1) showed that the sample size was sufficient for valid results (power > .80, *α* = .05, effect size *f*^*2*^ = 0.15).

### Measures

#### Positive Mental Health Scale

(PMH-Scale; original version: [6]). The PMH-Scale assessed subjective and psychological aspects of well-being across nine items (e.g., “I feel that I am actually well equipped to deal with life and its difficulties”) that are rated on a 4-point Likert-type scale (0 = *do not agree*, 1 = *tend to disagree*, 2 = *tend to agree*, 3 = *agree*). Higher scores indicate higher levels of PMH. Previous research reported a scale reliability of Cronbach’s *α* = .930 [6] for the German language PMH-Scale and *α* = .921 [11] for the English language PMH-Scale.

##### Translation of the PMH-Scale into the Persian language

In the present study, the PMH-Scale was translated into the Persian language by the customary translation-back-translation-modification procedure by three bilingual speakers of the Persian language and of the English language [27]: The first person translated the English language version of the PMH-Scale into the Persian language. Then, two independent persons translated the Persian language version back into the English language. The back-translations were compared with each other and with the English version by the first person. Considering the differences between the back-translations and the English version, this person modified the Persian version of the PMH-Scale. Next, the two independent translators translated again the modified Persian language version back into the English language. The process required overall three rounds of translation and back-translation before the Persian language version was judged to be satisfactory by all three translators.

#### Multidimensional Scale of Perceived Social Support

(MSPSS; original version: [28]; Persian version: [29]). Perceived social support from family, friends and significant others was assessed with the MSPSS. This instrument consists of twelve items (e.g., “There is a special person who is around when I am in need”) that are rated on a 7-point Likert-type scale (0 = *very strongly disagree*, 6 = *very strongly agree*; current scale reliability: *α* = .922). The higher the sum score, the higher the perceived social support.

#### Post-Traumatic Growth Inventory

(PTGI; original version: [30]; Persian version: [31]). Growth after traumatic events was measured with the PTGI that includes 21 items (e.g., “I changed my priorities about what is important in life”). The items belong to overall five domains (i.e., social connections, new possibilities, perceived skills and resources, life’s appreciation, spiritual beliefs) and are rated on a 6-point Likert-type scale (0 = *not at all*, 5 = *extremely*; current scale reliability: *α* = .937). Higher sum scores indicate higher post-traumatic growth.

#### PTSD Checklist

(PCL-5; original version: [32]; Persian version: [33]). The PCL-5 assessed symptoms of PTSD (i.e., intrusion, avoidance, cognitive and mood alteration, arousal and reactivity alteration) with 20 items (e.g., “In the past month, how much were you been bothered by: “Repeated, disturbing, and unwanted memories of the stressful experience?”) that are rated on a 5-point Likert-type scale (0 = *not at all*, 4 = *extremely*; current scale reliability: *α* = .939). The higher the sum score, the higher the sypmtoms of PTSD.

#### Patient Health Questionnaire

(PHQ-9; original version: [34]; Persian version: [35]). Depressive symptoms over the past two weeks were measured with the PHQ-9. The nine items (e.g., “Little interest or pleasure in doing things”) are rated on a 4-point Likert-type scale (0 = *not at all*, 3 = *nearly every day*; current scale reliability: *α* = .879). Higher sum scores indicate higher depressive symptpms.

#### Suicide Behaviors Questionnaire-Revised

(SBQ-R; original version: [36]; Persian version: [37]). The SBQ-R comprises four items assessing different aspects of suicidal ideation/behavior (lifetime suicide ideation, suicide plans, suicide attempts, 12-months suicide ideation, suicidal communication, and one’s estimation of how likely a future suicide attempt might be). Each item utilizes a different Likert scale with a sum score of 18 points indicating the highest severity of suicidal behavior. A cut-off score of 7 or higher has been established as an indicator for greater suicide risk [36]. The current scale reliability of the SBQ-R was *α* = .802.

### Statistical analyses

Statistical analyses were conducted using the Statistical Package for the Social Sciences (SPSS 26) and the macro Process version 3.5 (www.processmacro.org/index.html, [38]). After descriptive analyses, an exploratory factor analysis (EFA) using principal component analysis (PCA; rotation method: varimax) on the nine items that assessed PMH was calculated (see [39–41]). This step allowed to exploratorily investigate whether the unidimensional structure of the PMH-Scale that was reported by studies from other countries (e.g., Germany [6]) can be replicated in the present Iranian sample. Next, to investigate the construct validity of the PMH-Scale, its associations with social support and post-traumatic growth (convergent validity), as well as with depressive symptoms, PTSD and suicide ideation/behavior (discriminant validity) were assessed by the calculation of zero-order bivariate correlation analyses. Group differences in PMH scores between participants with greater suicide risk (SBQ-R ≥ 7) and participants with lower suicide risk (SBQ-R < 7) were tested via a t-test.

Finally, a moderation analysis (Process: model 1) examined the relationship between depressive symptoms (predictor), PMH (moderator) and suicide ideation/behavior (outcome), controlling for age and gender as covariates because of the mostly female and relatively young composition of the present sample. Following Hayes [38], the moderation effect was assessed by the bootstrapping procedure (10.000 samples) that provides percentile bootstrap confidence intervals (CI 95%).

## Results

Table 1 shows the descriptive statistics of the investigated variables.

**Table 1:** Descriptive statistics of the investigated variables and their correlation with positive mental health

	*M (SD)*	*Min–Max*	*r*
PMH-Scale	15.40 (6.18)	0–27	–
MSPSS	44.85 (16.69)	0–72	.460**
PTGI	58.99 (21.09)	3–105	.531**
PHQ-9	10.09 (6.47)	0–27	−.622**
PCL-5	28.91 (17.57)	0–95	−.489**
SBQ-R	5.30 (3.07)	3–18	−.451**

*N* = 573, *MSPSS* Multidimensional Scale of Perceived Social Support, *PCL-5* PTSD Checklist, *PHQ-9* Patient Health Questionnaire, *PMH-Scale* Positive Mental Health Scale, *PTGI* Post-Traumatic Growth Inventory, *SBQ-R* Suicide Behaviors Questionnaire-Revised, *M* Mean, *SD* Standard Deviation, *Min* Minimum, *Max* Maximum. **p < 001

### Factor analysis

In accordance with previous research (e.g., [6]), the EFA (Kaiser-Meyer-Olkin: KMO = .922; Barlett’s test of sphericity: χ^2^ = 2877.787, df = 36, *p* < .001) revealed a 1-factor structure of the PMH-Scale. The factor had an eigenvalue of 5.214 and explained 57.93% of the variance (cf., [41]). This finding confirmed the unidimensional structure of the PMH-Scale reported by previous research (e.g., [6, 8]).

### Scale properties

Internal consistency was assessed using Cronbach’s *α*. In the current study, it was *α* = .905. The mean interitem correlation was: *r*_mi_ = .517 (range: .285 to .783). The item-total scale correlation ranged between *r*_it_ = .426 (Item 1) and *r*_it_ = .813 (Item 2). The respectively deletion of single items provided no significant improvement of the internal consistency. The reliability ranged between *α* = .885 (deletion of Item 2) and *α* = .913 (deletion of Item 1). The item difficulty ranged between *p*_m_ = 45.1% (Item 1) and *p*_m_ = 63.4% (Item 9).

### Construct validity

As shown in Table 1, PMH was significantly positively correlated with social support and with post-traumatic growth (both: *p* < .001). Its correlation with depressive symptoms, PTSD and suicide-related outcomes was significantly negative (all: *p* < .001). Furthermore, participants with greater suicide risk (SBQ-R ≥ 7; *n* = 146, 25.5%) and participants with lower suicide risk (SBQ-R < 7, *n* = 427, 74.5%) differ significantly in PMH scores: SBQ-R ≥ 7: *M (SD)* = 11.22 (6.27), SBQ-R < 7: *M (SD)* = 16.83 (5.47), t (225) = 9.627, *p* < .001. These findings reveal the convergent and the discriminant validity of the Persian version of the PMH-Scale.

### Moderation analysis

**Fig. 1:**
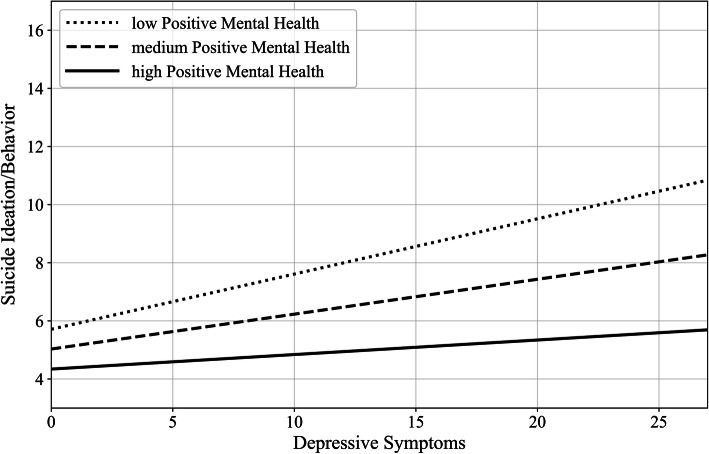
Moderation effect of positive mental health (moderator) on depressive symptoms (predictor) to suicide ideation/behavior (outcome)

The moderation model turned out to be statistically significant, *R*^2^ = .292, *F* (5,567) = 38.227, *p* < .001. The significant interaction between depressive symptoms and PMH, b = −.011, SE = .003, 95% CI [−.017, −.006], t = − 4.031, *p* < .001, revealed that the relationship between depressive symptoms and suicide ideation/behavior was moderated by PMH. According to the simple slopes tests, the positive link between depressive symptoms and suicide ideation/behavior was significant for low level (one *SD* below mean = − 6.182: b = .189, SE = .028, 95% CI [.133, .244], t = 6.673, *p* < .001) and medium level of PMH (mean = 0: b = .122, SE = .025, 95% CI [.073, .171], t = 4.897, *p* < .001). Notably, the link was stronger for low than for medium level of PMH. However, the link between depressive symptoms and suicide ideation/behavior was not significant for high level of PMH (one *SD* above mean = 6.182: b = .055, SE = .032, 95% CI [−.007, .116], t = 1.737, *p* = .083). Thus, PMH significantly moderated the association between depressive symptoms and suicide ideation/behavior. Specifically, the lower the PMH level, the closer the link between both variables. Figure 1 visualizes the moderation effect.

## Discussion

In the present study, the reliability and construct validity as well as the postulated factor structure of the Persian version of the Positive Mental Health Scale (PMH-Scale) were investigated. In line with the original version of the PMH-Scale [6] and its various translated versions [8, 9, 11], the Persian version of the PMH-Scale had a unidimensional factor structure and an excellent internal consistency. These results confirm previous findings from other countries (e.g., [6, 11] that described the PMH-Scale as a time-efficient instrument for the assessment of positive mental health. Moreover, they extend the research on PMH by providing a valid and reliable Persian language version of this instrument that can be applied in Iranian samples.

Construct validity of the PMH-Scale was supported by expected associations between the PMH-Scale and depressive symptoms, suicide ideation/behavior and social support (cf., [6, 19]). The finding on significant associations between PMH scores and trauma-related constructs (i.e., post-traumatic symptoms and post-traumatic growth) extends prior work on the PMH and complements findings from a Lithuanian study showing a negative association between PMH, life stressors and adjustment disorder symptoms [9]. Taken together the present results underscore the relevance of PMH in relation to a broad spectrum of psychopathological symptoms. A possible reciprocal relationship between PMH and post-traumatic growth should be considered in more detail in future studies (cf., [42]).

PMH scores differentiated between participants with higher vs. lower suicide risk. Furthermore, PMH moderated the association between depressive symptoms and suicide ideation/behavior. Therefore, it was possible to replicate a finding previously shown in samples of German and Chinese students [19]: Those participants who reported a greater level of PMH were less likely to experience suicide ideation/behavior even at the highest severity of depressive symptoms as compared to participants who reported low level of PMH. This is yet another indication that PMH can be considered as conferring resilience (cf., [43]).

In terms of practical implications, the results of the current study underscore the importance of accounting for the presence of PMH in addition to aspects of psychopathology. Furthermore, the fact that PMH can significantly alter the impact of depressive symptoms on suicide ideation/behavior may be an important aspect to incorporate in clinical interventions. Finally, preventive programs for student populations may benefit from a focus on the fostering of PMH. Notably, first available studies showed that physical activity and loving kindness meditation have a positive impact on PMH [44, 45]. With respect to the fact that self-acceptance and environmental mastery are central facets of PMH [21], clinicians might also focus on fostering self-compassion [46] and (renewed) access to personal strengths and resources [47, 48].

Several limitations have to be considered when interpreting the current results. First, since 100% of the sample were students, it is unclear how the findings would generalize to a more diverse and/or clinical population. Yet, with regard both to suicide ideation/behavior [25] as well as changes in well-being [49] student populations are a group of special concern. Second, the cross-sectional design of the current study precludes analyses of test-retest reliability and scalar invariance over time. Longitudinal studies on these issues are warranted. In a similar vein, it is necessary to investigate, whether PMH does not only buffer the impact of depressive symptoms on suicide ideation/behavior in a cross-sectional study design, but also in a longitudinal study design (cf., [24]). Third, the current study utilized only self-report measures of depressive symptoms, post-traumatic symptoms, and suicide ideation/behavior. This method has certain advantages, for example, the measures are economical and easy to administer. However, self-report measures may fail to capture suicide ideation/behavior, depressive symptoms, or post-traumatic symptoms in their full complexity. Fourth, to establish convergent validity, it would have been good if other measures of PMH, such as the Psychological Well-Being Scale [50] or the Satisfaction with Life Scale [51], had been available. Fifth, when investigating the factor structure of a scale, an exploratory factor analysis is recommended to be complemented by a confirmatory factor analysis. Both analyses should be calculated with independent samples [39–41]. In the present study, only one sample has been assessed that was used for the exploratory factor analysis. Therefore, future research is advised to further investigate the factor structure of the current Persian version of the PMH-Scale by the calculation of a confirmatory factor analysis in a separate sample. Moreover, the current findings indicate that the cultural invariance of the Persian version of the PMH-Scale can be determined in a next step by the comparison of Iranian data with data gained in other countries [8, 42].

## Conclusion

Despite some limitations, the current results suggest that the Persian version of the PMH-Scale is a brief, reliable, and valid measure of subjective and psychological well-being that can be used to complement mental health assessments in research and practice.

## Data Availability

All relevant data are reported within the paper. Analyzed data are available from the corresponding author on reasonable request.

## Abbreviations

MSPSS: Multidimensional Scale of Perceived Social Support
PCL-5: PTSD Checklist
PHQ-9: Patient Health Questionnaire
PMH: Positive Mental Health
PTGI: Posttraumatic Growth Inventory
SBQ-R: Suicide Behaviors Questionnaire-revised
